# Difference of Success Rates of Mineral Trioxide Aggregate Pulpotomies Performed Both by Undergraduate Dental Students and by an Expert Operator: A Retrospective Study

**DOI:** 10.1155/2017/4385423

**Published:** 2017-08-24

**Authors:** Marco Pasini, Maria Rita Giuca, Roberto Gatto, Silvia Caruso

**Affiliations:** ^1^Department of Surgical, Medical, Molecular Pathology and Critical Area, Unit of Pediatric Dentistry, University of Pisa, Pisa, Italy; ^2^Department of Life, Health and Environmental Sciences, University of L'Aquila, L'Aquila, Italy

## Abstract

**Aim:**

The aim of this retrospective study was to evaluate the clinical and radiographic success of pulpotomy on primary molars performed by dental students compared to that performed by an expert operator.

**Methods:**

The study was conducted on 142 second primary molars in 102 children. The patients were randomly selected from the available records. The test group (treated by dental students) included 51 subjects (28 males and 23 females, mean age: 7.2 ± 1) and the control group included 51 children (29 males and 22 females, mean age: 7.4 ± 1.2 years). After pulpotomy, a clinical and radiographic evaluation after 12 months was performed. Chi-square test and odds ratio were calculated and significance level was set at *p* < 0.05.

**Results:**

The success rate was significantly lower, 81.6% (*p* < 0.05), in the test group than in the control group (93%). The test group showed less clinical and radiographic success (86% and 80%, resp.) compared to the control group (97.2% for clinical success and 93% for radiographic success).

**Conclusions:**

Pulpotomy with MTA is an effective method that ensures a good percentage of success. The clinical experience of the operator is a contributing factor.

## 1. Background

Pulpotomy is a therapeutic procedure, frequently used in paediatric dentistry, which aims at eliminating the pulp from the pulp chamber while maintaining the vitality of the root pulp [1].

Before performing it, patient history is necessary in order to exclude the presence of spontaneous pain; in addition, sensitivity to percussion or to palpation should be absent, with positive response to vitality tests.

Pulpotomy is also contraindicated in the presence of swelling, fistula, pathological mobilization, internal root resorption, pulp calcifications, or excessive bruising of the root pulp [2].

It can be performed in case of exposure of the vital pulp in presence of a sufficient rooting structure and in absence of periradicular pathologies that may affect the permanent teeth still to be erupted.

It can also be performed on permanent teeth but only as emergency intervention until complete endodontic therapy can be performed or as a temporary intervention on permanent teeth with immature root formation to allow it to be developed [3].

The partial removal of carious dentin is the currently indicated technique in extensive caries lesions. In dentinal cavitated caries lesions that radiographically appear to extend less than 75 percent into the dentin, this technique is often used without the risk of exposing the pulp [4].

Compared to the deciduous tooth extraction, the pulpotomy has the advantage of keeping the dental element in its arches up to its natural exfoliation with the following advantages: maintaining the guiding function of the teeth for the underneath permanent teeth in eruption, maintaining a better chewing and aesthetic function, and maintaining arch space (important in the case of the second primary molars in order to maintain the lee-way space and avoid the mesialization of the permanent molar). In addition, compared to the surgical therapy, it represents a more conservative method generally more accepted by both children and parents [5].

Compared to pulpectomy, it is a more conservative procedure and it is easier to perform because root canals of deciduous teeth are often more difficult to treat compared to permanent ones.

After removing the pulp chamber tissue by using a manual excavator or a round diamond bur, the hemostasis control is carried out, and then there is the application of a material at the level of the root canal entry in order to maintain the vitality of the root pulp, before proceeding with the tooth restoration.

There are many materials that can be used, such as formocresol, calcium hydroxide, ferric sulfate, and biodentine [6].

In addition, electrocautery and removal of the pulp by laser techniques have the advantage of controlling bleeding, although there is weak evidence of tissue repair.

Among the most commonly used materials for pulpotomy, there is the MTA (mineral trioxide aggregate) introduced in 1995, made of Portland cement (75%), bismuth oxide (20%), and calcium dihydrate sulfate (5%). Dicalcium and tricalcium silicate react with water causing, after hardening, the formation of a crystalline matrix with the formation of a dentinal bridge [7].

It has excellent sealing ability, and there are bone morphogenic proteins and growth factors that act through their osteogenic potential in pulp repair.

It has a bacteriostatic action, having a pH between 10.2 and 12.5; it is insoluble and acts in the presence of a humid environment.

Among the disadvantages associated with the use of the MTA, there are the high cost, the difficulty of removal for the check of the formation of the dentine bridge, and the discoloration at the dental level [6].

There is no unanimous agreement in the literature on which material is actually the best, despite the fact that many studies seem to show a slight improvement in pulpotomy performed with MTA compared to other materials [5, 8, 9].

However, besides the material, it is essential to diagnose and to proceed correctly in every step of the therapy.

Since all pulpotomy phases must be performed correctly in order to achieve good therapeutic results, success also may depend on the operator's experience.

There are many studies in literature comparing the clinical and radiographic successes of pulpotomy-treated teeth, while fewer studies investigated the success of treatment with reference to operator's experience.

In a study conducted by Odabaş et al. (2012), the clinical and radiographic success rate of pulpotomy performed with two different materials (mineral trioxide aggregate and ferric sulfate) by university students was evaluated, showing slightly lower values (94.7% for clinical success and 92.1% for radiographic success with MTA) compared to data obtained for more expert operators [10].

Therefore, the aim of the present study is to evaluate the clinical and radiographic success of pulpotomies carried out with the same materials by an expert operator compared to those performed by the students of the last year of the dental school [11].

## 2. Materials and Methods

### 2.1. Study Population

In this retrospective study, clinical records of paediatric patients between 6 and 9 years of age were analyzed at the university clinic.

The teeth considered were only the mandibular second primary molars in patients in good general health condition, needing a pulpotomy due to exposure of the pulp, after caries removal. Patient records included clinical and radiographic evaluations that were performed by an operator different from the one who performed the therapies. The exclusion criteria were bleeding from root canals lasting more than 5 minutes (with cotton soaked in sterile saline pellets), spontaneous pain or pain at percussion, pathological mobility and swelling, presence of infiltration or failure of restoration, performed at the end of pulpotomy, and presence of incomplete clinical records.

In addition, the teeth had to lack internal or external root resorption or destruction of the periradicular bone tissue according to radiography (endoral radiographs).

The local ethics committee approved this study.

The sample size was calculated by considering the clinical success of pulpotomy, performed with MTA, found in previous studies both by operators with little experience [10] and by expert operators [3].

The sample size calculation resulted in an 80% power at a 5% level of statistical significance and a 10% of difference between the groups, requiring 71 teeth for each group.

The study was conducted on a total of 142 second primary molars (71 molars in the test group and 71 molars in the control group) in 102 children (57 males and 45 females, mean age of 7.3 ± 1.1. years, ranging from 6 to 9 years). The patients were randomly selected and a random computerized analysis was performed to select the patients from a pool of available patient records treated by dental students and a pool of patients records treated by a paediatric dentist with more than 10 years of experience in this field.

The test group (treated by dental students) included 51 subjects (28 males and 23 females, mean age: 7.2 ± 1) and the control group (treated by an expert dentist) included 51 children (29 males and 22 females, mean age: 7.4 ± 1.2 years).

### 2.2. Clinical Procedures

All the operators carried out the pulpotomy with the same tools and in the same place. Test group operators were 10 students of the last year of dental school who had undergone preliminary training on pulpotomy procedures on extracted teeth but who had never performed this type of therapy on patients. The therapies were performed under the supervision of a tutor and with the help of another student as an assistant.

The procedure was performed by starting with a mandibular nerve block and a rubber dam isolation was placed. Caries excavation was performed with a round diamond bur (#6), at high speed under water-spray cooling. The surface of the remaining pulp was irrigated with sterile water. Excessive air on the exposed pulp, which may cause tissue desiccation, was avoided.

Then, MTA-Angelus (Angelus, Londrina, PR, Brazil) was applied at the entrance of the root canal, following instructions in order to obtain a putty-like consistency. The mixture was applied to the pulp stumps and condensed lightly with a cotton pellet to obtain a thickness of 2 mm.

Definitive restorations consisted of glass ionomer cement (Fuji Lining LC, GC) as a liner and were completed with composite resin (Enamel TM, Micerium).

Radiographic evaluation was performed by digital intraoral X-rays (Kodak 2100) with the parallel ray technique, Rinn centring, and a 2x magnification viewer.

Clinical success was the absence of spontaneous pain, tenderness at percussion, swelling, and pathologic mobility. In addition, the radiological parameters of success were the absence of exfoliation, flaring of the periodontal ligament space, internal or external root resorption, and radicular radiolucency. The presence of at least one of these dental signs was considered as a failure of the therapy.

Parameters have been used at the beginning of the treatment (T0) and after 12 months (T1).

At the end of the follow-up, the number of teeth which, even after being treated with pulpotomy, still required extraction was calculated.

### 2.3. Statistical Analysis

Statistical analysis was performed by using SPSS 22.0 (SPSS Inc., Chicago, IL, USA) and the level of significance was set at *p* < 0.05.

Chi-square test, odds ratio (OR), and confidence interval (CI) were calculated to evaluate the intragroup differences (from T0 to T1) and intergroup differences between test and control groups. All radiographs were evaluated twice after 2 weeks, and the kappa score was 0.88.

## 3. Results

The results of this retrospective study are shown in Table 1.

A significant difference (*p* < 0.05) was found in the test group for both clinical and radiographic success, from the beginning of therapy up to 12 months. Similarly, a significant difference (*p* < 0.05) was found in the control group at the end of the follow-up for radiological and total success. However, no significant intergroup differences have been reported during clinical controls from the beginning to the end the treatment (*p* > 0.05).

Patients in the test group showed a total percentage of therapeutic successes after pulpotomy statistically lower than the control group (*p* < 0.05).

In particular, as far as clinical success is concerned, the group of patients treated by an expert operator has achieved almost a 100% success, while in the group of children treated by a less expert operator, however, a good clinical but statistically inferior success was achieved (*p* < 0.05).

Regarding the radiological success, a statistically significant difference (*p* < 0.05) was observed between the two groups, with a greater percentage in the control group than the test group.

In the group of children treated by an expert operator, however, 5 teeth showed both clinical and radiographic failure, while two molars showed only a clinical failure.

Clinical record data reported that, in the test group, 6 teeth were extracted and 4 teeth were treated with pulpectomy in the 12-month follow-up.

However, in the control group, 2 teeth were extracted while no tooth was treated with pulpectomy.

## 4. Discussion

The results of the present study showed that pulpotomy is an effective therapeutic method that can be performed on primary molars, in order to ensure good long-term success.

Since it consists of several steps, it is crucial to have a proper diagnosis and to carry out all procedural steps correctly [12]. An additional difficulty may be represented by the paediatric patient, who usually shows less collaboration than the adult and is unable to perform long and tiring therapies.

The present study showed that the experience is an important factor to be considered as it seems to ensure a better therapeutic success, for the same material.

In a recent study conducted in a survey of 51 paediatric dental schools in 22 different European universities, the most commonly used material is MTA (taught and used in 37 schools), followed by ferric sulfate that is taught in 29 dental schools. In addition, in most dental schools, pulp treatment in deciduous teeth is taught to both undergraduate and postgraduate students [13].

Clinical and radiographic therapeutic successes obtained in the present study confirmed those of the literature, although showing slightly lower success rates.

In particular, as far as pulpotomy performed by expert operators is concerned, the total failure rate obtained with MTA is, in most of the study, below 10%, after 12 months.

In the study conducted by Godhi and Tyagi (2016) on 25 primary molars, a 100% clinical success and a 96% radiological success were observed after a 12-month follow-up [12].

Moreover, in a study conducted by Eidelman et al. (2001), the success of pulpotomy with MTA was 100% [14].

The results of the control group were similar to those found by Srinivasan and Jayanthi, who showed a 95.7% radiologic success rate of pulpotomy after 12 months [15].

However, the percentage of success in children treated by dentistry students was lower, despite the supervision of the tutor.

One possible explanation for the high failure rate may be because dentistry students had never performed this treatment directly on the patient before and therefore several factors may have affected it (fear, difficulties in clinical evaluation, and manual difficulty). Compared to treatments performed on extracted teeth, the treatments performed directly on the patient are more difficult due to the presence of saliva, blood, and the difficulty of approaching a paediatric patient, even from a psychological point of view.

In a study conducted by Honey et al. (2011), it has been observed that senior dentistry students are more confident with the simple therapeutic procedures such as application of sealants and scales and polish, while having a low self-reported confidence for more complex performances, such as extraction [16].

A significant correlation among clinical experience levels and exam score was observed in dentistry students, as far as paediatric dentistry is concerned; in the Cork University Dental School and Hospital of Ireland, undergraduate students gained experience in management of paediatric patients with students providing care for an average of thirty children and a minimum of nineteen [17].

In a study conducted by Henzi et al. (2006) on 655 junior, senior, and graduate dental students in twenty-one North American dental schools, it was observed that dental school clinic was often an inefficient learning environment that hindered their opportunity to develop clinical competency [18].

Especially in modern dentistry, due to the vastness of different materials and techniques, it is very difficult to reach enough clinical experience in all major fields of dentistry before graduation.

The evaluation in academic environments should reflect the learning outcomes of the training and must meet the following key objectives: assessing the learning process with a feedback system and evaluating attitudes and skills, such as critical thinking, self-assessment capacity, and manual and clinical skills [19].

Among the possible techniques aimed at improving clinical learning, in addition to the internship, literature has highlighted the effectiveness of case-based teaching (clinical case presentation), which allows developing analytical reasoning, through discussion of real clinical cases [20].

It has also been noted that modern teaching methods that use digital technology and digital simulations represent a significant potential for dental education and enable faster acquisition of some operational abilities [21].

The limitations of the study included the absence of a short-term follow-up; for this reason, we are not exactly able to detect failures in the first 12 months. An additional follow-up, after 1 year, could be useful in order to evaluate the success rates in the following period. Our analysis is limited to MTA; however other different materials, such as calcium hydroxide and ferric sulfate, could be included for further investigations in order to compare the clinical and radiographic success rates.

Further studies will be necessary to confirm the results of the present study.

## 5. Conclusions

The results of the present study demonstrate that pulpotomy performed on primary molars with MTA represents a high clinical and radiographic success rate method after a 12-month follow-up.

Considering the experience of the operator, it was noted that the group of patients treated by less experienced operators (dental students) showed less clinical and radiographic success compared to the group treated by an expert operator.

## Figures and Tables

**Table 1:** Success of the two groups after 12 months.

	Test group (12 months)	Control group (12 months)	Intragroup test *p* value	Odds ratio (CI)	Intragroup control *p* value	Odds ratio (CI)	Intergroup *p* value	Odds ratio (CI)
Total success	*N*: 58 (81.6%)	*N*: 66 (93%)	0.001	0.45 (0.37–0.54)	0.02	0.48 (0.4–0.57)	0.04^*∗*^	0.34 (0.11–1.01)
Clinical success	*N*: 61 (86%)	*N*: 69 (97.2%)	0.001	0.46 (0.38–0.56)	0.15	0.49 (0.42–0.58)	0.01^*∗*^	5.7 (1.19–26.8)
Radiographic success	*N*: 57 (80%)	*N*: 66 (93%)	0.001	0.45 (0.37–0.54)	0.02	0.48 (0.4–0.57)	0.02^*∗*^	3.24 (1.1–9.6)

^*∗*^ *p* < 0.05.
